# Integrating Dimension Reduction and Out-of-Sample Extension in Automated Classification of Ex Vivo Human Patellar Cartilage on Phase Contrast X-Ray Computed Tomography

**DOI:** 10.1371/journal.pone.0117157

**Published:** 2015-02-24

**Authors:** Mahesh B. Nagarajan, Paola Coan, Markus B. Huber, Paul C. Diemoz, Axel Wismüller

**Affiliations:** 1 Departments of Imaging Sciences and Biomedical Engineering, University of Rochester Medical Center, Rochester, New York, USA; 2 Faculty of Medicine and Institute of Clinical Radiology, Ludwig Maximilian University, Munich, Germany; 3 Faculty of Physics, Ludwig Maximilian University, Munich, Germany; 4 European Synchrotron Radiation Facility, Grenoble, France; University of Nebraska Medical Center, UNITED STATES

## Abstract

Phase contrast X-ray computed tomography (PCI-CT) has been demonstrated as a novel imaging technique that can visualize human cartilage with high spatial resolution and soft tissue contrast. Different textural approaches have been previously investigated for characterizing chondrocyte organization on PCI-CT to enable classification of healthy and osteoarthritic cartilage. However, the large size of feature sets extracted in such studies motivates an investigation into algorithmic feature reduction for computing efficient feature representations without compromising their discriminatory power. For this purpose, geometrical feature sets derived from the scaling index method (SIM) were extracted from 1392 volumes of interest (VOI) annotated on PCI-CT images of ex vivo human patellar cartilage specimens. The extracted feature sets were subject to linear and non-linear dimension reduction techniques as well as feature selection based on evaluation of mutual information criteria. The reduced feature set was subsequently used in a machine learning task with support vector regression to classify VOIs as healthy or osteoarthritic; classification performance was evaluated using the area under the receiver-operating characteristic (ROC) curve (AUC). Our results show that the classification performance achieved by 9-D SIM-derived geometric feature sets (AUC: 0.96 ± 0.02) can be maintained with 2-D representations computed from both dimension reduction and feature selection (AUC values as high as 0.97 ± 0.02). Thus, such feature reduction techniques can offer a high degree of compaction to large feature sets extracted from PCI-CT images while maintaining their ability to characterize the underlying chondrocyte patterns.

## Introduction

Osteoarthritis (OA) is now established as one of the leading causes of disability worldwide [1–3]. This disease is characterized by loss of articular cartilage, thickening of the underlying subchondral bone, and osteophyte formation [4]. Given that monitoring OA progression for purposes of patient health evaluation and response-to-therapy assessment are currently of significant interest, it would be desirable to have an imaging modality that could provide early detection and visualization of any degenerative modifications to cartilage [5–10]. Several imaging techniques are currently under investigation for their ability to assess cartilage health, eg. delayed gadolinium-enhanced MR imaging of cartilage (dGEMRIC) [11], ^23^Na MRI [12], T1*ρ* [13], GAG chemical exchange saturation transfer (gagCEST) [14] etc. These techniques focus on quantifying cartilage matrix composition where changes in water and collagen content, and loss in glycosaminoglycan (GAG) content have been previously identified as early signs of cartilage degeneration [13].

In this context, phase contrast X-ray computed tomography (PCI-CT) has recently emerged as a novel imaging modality that can visualize the internal architecture of the cartilage matrix at micro-meter scale resolution. Rather than rely on bio-chemical markers, PCI exploits the phase contrast effect associated with X-ray refraction in soft tissue, which is more pronounced than conventional absorption contrast in cartilage, as previously shown in [15]. Analysis of PCI-CT images acquired from ex vivo patellar cartilage specimens highlighted differences in chondrocyte organization between healthy and osteoarthritic cartilage matrix. Specific differences were noted in the radial zone where healthy specimens exhibited chondrocyte alignment (known as Benninghoff’s arch [16]) while osteoarthritic specimens presented disorganized chondrocyte clustering throughout the matrix [15]. The high spatial resolution afforded by PCI-CT enables the use of texture analysis methods based on statistics (gray-level co-occurrence matrices or GLCM), topology (Minkowski Functionals), geometry (Scaling Index Method), etc to characterize these differences, as pursued in previous studies [17, 18]. Such textural approaches provide quantitative measures that could potentially serve as imaging markers for detecting and quantifying OA-induced degenerative changes to the cartilage matrix.

Textural approaches involving topological or geometrical features, as outlined in previous work [17, 18], provide a detailed characterization of the cartilage matrix through extraction of large feature sets. However, the extraction of too many features poses problems as it can contribute to overall deterioration in classification performance of machine learning algorithms, referred to as the so-called curse of dimensionality [19]. It has also been suggested that irrelevant or noisy features can adversely affect classification performance. Such problems highlight the need for employing some form of feature reduction to obtain an efficient representation of the original feature space while simultaneously maintaining adequate separability between the two classes of patterns, i.e. healthy and osteoarthritic. Feature reduction has been previously achieved with feature selection algorithms in the context of lung [20], breast [21, 22] etc. In such approaches, the original feature set is reduced through explicit exclusion of features either redundant in information content or irrelevant to the classification task. More recently, dimension reduction has also been proposed as an alternative to feature selection in the context of breast lesion classification [23–25]. Dimension reduction allows for an algorithmic weighting of all features in the original set while computing a newer smaller feature set.

We are specifically focused on evaluating the classification performance of feature sets extracted from post-processed phase contrast X-ray CT data for purposes of establishing imaging markers that can quantify OA-affected cartilage tissue. To that end, this specific study aimed at analyzing the impact of incorporating feature reduction on the performance achieved with previously proposed geometrical feature sets [18] in classifying healthy and osteoarthritic cartilage tissue on PCI-CT. For this purpose, we present a new CADx methodology in this work where dimension reduction is integrated in conjunction with out-of-sample extension. Dimension reduction is applied to the training subset of the feature sets extracted from patellar cartilage VOIs; corresponding low-dimension representations for the test set are computed using out-of-sample extension techniques. Thus, a strict separation between training and test sets is maintained in our methodology, which is crucial for the supervised learning step in such automated classification tasks. This separates our work from previous attempts described in [23, 24] where dimension reduction was applied to the entire dataset prior to machine learning, which violates this training-test separation requirement. Our improved CADx methodology is described in detail in the following sections.

## Data

### Patellar Cartilage Samples

Age of the donor, macroscopic visual inspection and probing of the cartilage surface at autopsy were taken into account for selection of patellae. Donors older than 40 years were a priori excluded for harvest of normal samples while no constraint in age was imposed on potential donors for osteoarthritic samples. A smooth, white, and shiny surface present across the entire patellar cartilaginous surface and prompt resilience to manually performed focal indentation probing were criteria that defined macroscopically normal cartilage. Lack of these criteria in addition to visually perceived defects in the joint surface were used to select osteoarthritic samples. Based on these inclusion criteria, 2 healthy and 3 osteoarthritic cylinder-shaped osteochondral samples (diameter: 7 mm) were extracted within 48 hours postmortem from the lateral facet of 4 human patellae using a shell auger. Cylinders were trimmed to a total height of 12 mm including the complete cartilage tissue and the subchondral bone. The samples were continuously rinsed by 0.9% saline during extraction, trimming and removal of soiling from sawing. During image acquisition, samples were dipped into a 10% formalin solution.

### PCI Experimental Setup

The image acquisition used the analyzer-based imaging (ABI) PCI technique, which has been previously demonstrated as highly sensitive to small phase variations [26]. The setup consisted of a parallel quasi-monochromatic X-ray beam, used to irradiate the sample, and of a perfect crystal, the analyzer, placed between the sample and the detector [27]. The analyzer acted as an angular filter of the radiation transmitted through the object and only the X-rays traveling in a narrow angle range close to the Bragg condition were diffracted onto the detector. Before being detected, the beam was modulated by the angle-dependent reflectivity of the crystal (rocking curve), which had a full width at half maximum (FWHM) typically of the order of a few micro-radians. All images were acquired at the half maximum position on one slope of the rocking curve (50% position), which was chosen to achieve the best sensitivity. Further details of this ABI technique can be found in [15, 28].

Experiments were performed at the Biomedical Beamline (ID17) of the European Synchrotron Radiation Facility (ESRF, France). Quasi-monochromatic X-rays of 26 keV were selected from the highly collimated X-ray beam by means of a double Si (111) crystal system and an additional single Si (333) crystal [29]. The emerging refracted and scattered radiation from the sample was analyzed with a Si (333) analyzer crystal. The imaging detector used was the Fast Readout Low Noise (FReLoN) CCD camera developed at the ESRF [30]. The X-rays are converted to visible light by a 60 *μ*m thick Gadox fluorescent screen; this scintillation light is then guided onto a 2048×2048 pixel 14×14 m^2^ CCD (Atmel Corp, US) by a lens-based system. The effective pixel size at the object plane was 8×8 *μ*m^2^.

### Tomographic Image Reconstruction

**Fig 1:**
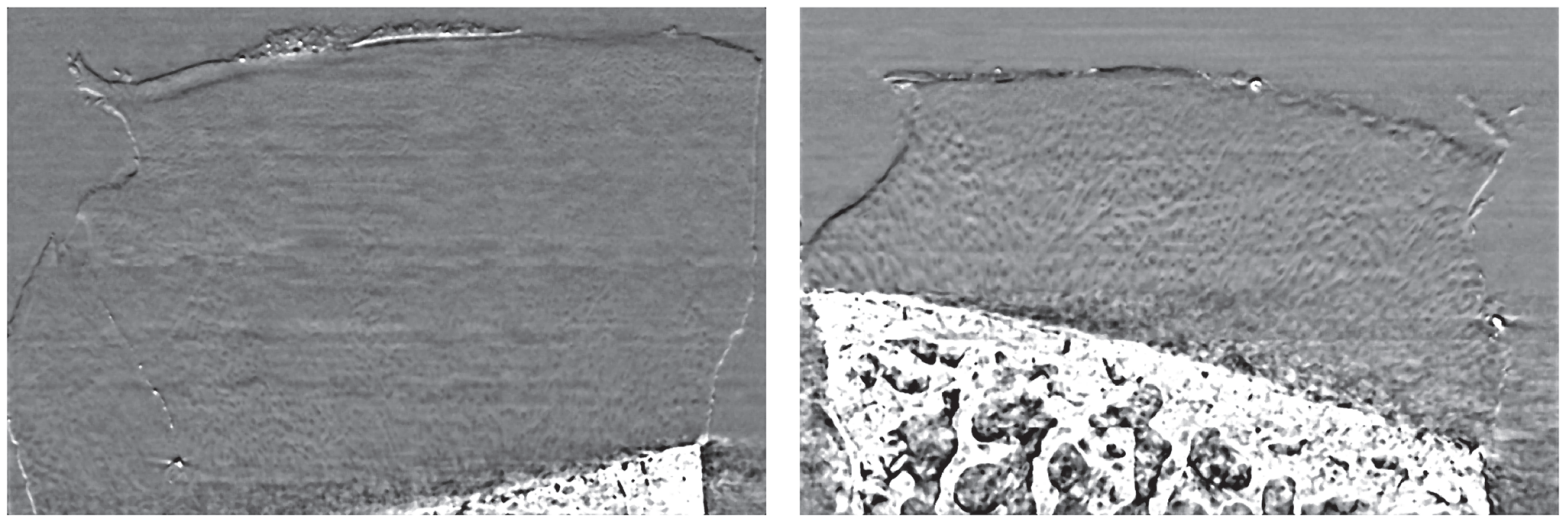
Coronal reconstruction created from axial PCI-CT images of a healthy (left) and osteoarthritic (right) cartilage specimens. Our interest is in the radial zone where chondrocyte organization is distinctly different in these two specimens.

In order to acquire tomographic images with our PCI experimental setup, the cartilage samples were rotated about an axis perpendicular to the incident laminar beam. At the end of each rotation, the sample was displaced along this axis to enable imaging of a different region. Unlike conventional CT imaging, the beam and detector were kept stationary. To reduce the effects of any spatial and temporal X-ray beam inhomogeneities, we then performed a flat field normalization for each angular projection image. A direct Hamming filter backprojection (FBP) algorithm was used for reconstructing tomographic images [31]. For data analysis, coronal slices were reconstructed from the original data and subject to edge-preserving median filtering with a [5 5 5] sliding window to smoothen noise artifacts. An example image acquired from one healthy and one osteoarthritic specimen is shown in Fig. 1.

### Pattern Annotation

Chondrocyte patterns were annotated with 3D cubic volumes of interest (VOI) in the radial zone of the cartilage matrix on the reconstructed PCI-CT images of all five specimens. 842 VOIs were annotated in total, of which 455 were osteoarthritic and 387 were healthy. The annotations were made using a cube of 25×25×25 pixels; the choice of VOI size was determined empirically based on previous work [18].

## Methods

### Ethics Statement

The institutional review board (IRB) of the Ludwig Maximilian University, Munich, Germany waived the need for ethical approval for this study since it involved retrospective analysis of anonymized tissue samples and imaging data collected from donors postmortem.

### Overview

**Fig 2:**
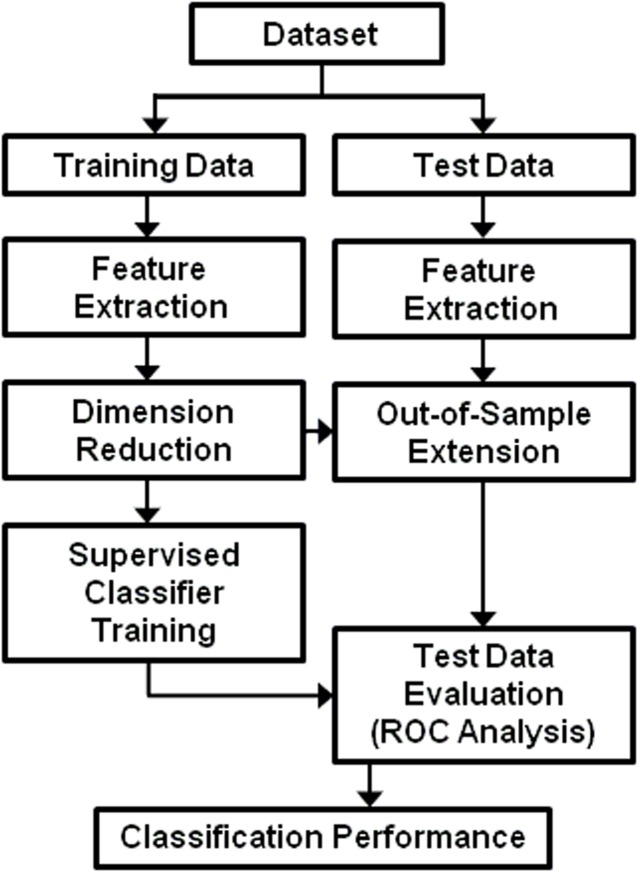
An overview of our CADx methodology proposed in this study. Our proposed methodology limits the application of dimension reduction to the training data alone, thus preserving the integrity (independence) of the test set. Low-dimension representations for the test set are obtained through out-of-sample extension.

Fig. 2 presents the CADx methodology proposed and evaluated in this study. Different components of this system are described in this section.

Feature extraction is achieved with the use of novel geometrical features derived from the Scaling Index Method (SIM). While originally developed for analyzing multi-dimensional arbitrary point distributions through evaluation of the surrounding structural neighborhood [32], SIM has since been extended for estimating local scaling properties (or local dimension) of the gray-level intensity map within an annotated VOI [33]. In this work, texture analysis using SIM is pursued because of its suitability to the task of classifying between healthy and osteoarthritic cartilage, as previously demonstrated in [18].

The extracted feature vectors are then separated into training and test sets. The high-dimension feature vectors in the training set alone are subject to dimension reduction. While a wide variety of dimension reduction algorithms are described in the literature, we showcase our CADx methodology by focusing on a balanced selection of dimension reduction techniques (with respect to algorithmic properties) principal component analysis or PCA (non-parametric, linear) [34], Sammon’s mapping (classical gradient descent, non-linear) [35], t-distributed stochastic neighbor embedding or t-SNE (global optimization, non-linear) [36] and exploratory observation machine or XOM (local optimization, non-linear) [37]. The corresponding low-dimension representations for the test set are obtained using out-of-sample extension techniques. In particular, we investigate the use of Shepard’s interpolation [38] and function approximation with a generalized radial basis function neural network (GRBF-FA) [39]. For comparison with dimension reduction, feature selection through evaluation of mutual information criteria [20] is also used.

Feature reduction is followed by supervised learning and classification, which is achieved through support vector regression (SVR) [40]. These processing steps were used to evaluate the classification performance achieved with our proposed CADx methodology of maintaining training-test data separation while applying dimension reduction. Individual components of this system are described in further detail in the following sub-sections.

### Texture Analysis

**Fig 3:**
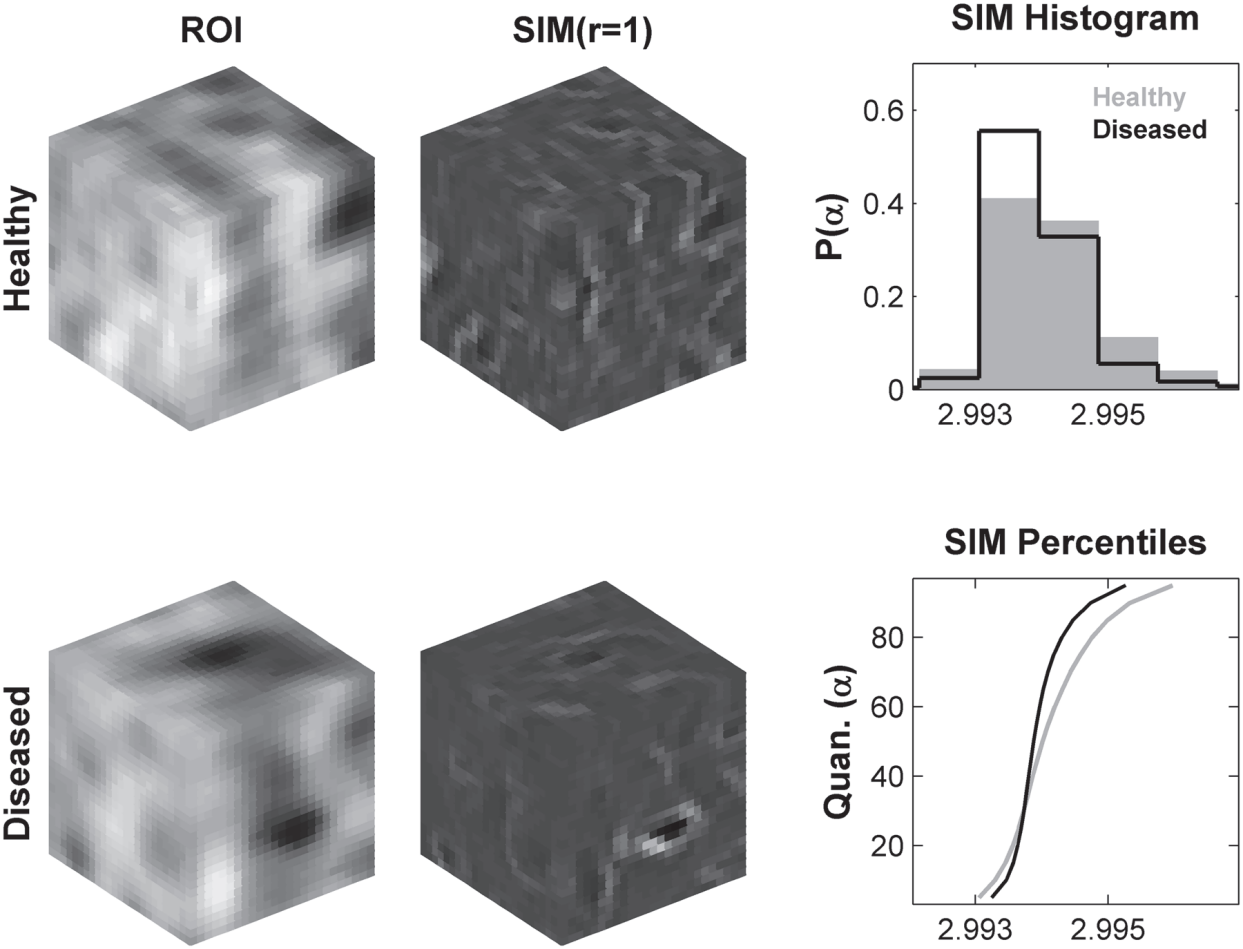
An illustration of the SIM feature extraction process. Examples of a normal and osteoarthritic VOIs (left), and their corresponding SIM transformations for radius *r* = 1 (middle). In the SIM transformations, dark regions correspond to lower magnitudes of *α* while brighter regions reflect higher magnitudes of *α*. The distribution of *α*-values from each SIM transformation are represented by histograms (top right) and by 9 percentiles (10^*th*^–90^*th*^) (bottom right).

In a specific VOI, all *N* voxels are represented by a 4-D vector *x*_*i*_ = (*x*_*i*_, *y*_*i*_, *z*_*i*_, *g*_*i*_), *i* = 1, 2, … *N*, consisting of three spatial dimensions (*x*_*i*_, *y*_*i*_, *z*_*i*_) and voxel gray-level intensity *g*_*i*_ = *g*(*x*_*i*_, *y*_*i*_, *z*_*i*_). A unit scaling constant was used to define the relationship between the spatial and intensity dimensions of each voxel. The application of SIM for a given scale *r* can be treated as an image transformation where each voxel within the VOI is assigned a local scaling property *α*_*i*_ = *α*(*x*_*i*_, *r*). This scaling property reflects the structural and geometrical properties of the surface formed in the voxel neighborhood defined by *r*. We use a previously proposed estimator for *α* that uses a Euclidean distance metric and Gaussian shaping functions, i.e.,

$$\begin{array}{c} \alpha \left(x_{i},r\right)=\frac{2\sum_{j=1}^{N}{\left(d_{ij}/r\right)}^{2}e^{-{\left(d_{ij}/r\right)}^{2}}}{\sum_{j=1}^{N}e^{-{\left(d_{ij}/r\right)}^{2}}}, \end{array}$$ (1)

where *d*_*ij*_ is the Euclidean distance between pixel *x*_*i*_ and neighboring pixel *x*_*j*_ and *r* is the radius of the Gaussian neighborhood [33]. After the SIM transformation is computed, the resulting distribution of *α*-values reveals non-linear structural information of the gray-level patterns annotated in the VOI. Nine quantiles (10^*th*^, 20^*th*^…90^*th*^) of this distribution were computed and used as a 9-D geometrical feature vector. The neighborhood radius was fixed as *r* = 1 based on previous work [18]. This is further illustrated in Fig. 3 using PCI-CT VOI examples.

### Feature Reduction—Dimension Reduction

The goal of dimension reduction in this study was to obtain low-dimension representations of high-dimension feature vectors for subsequent classification. Specifically, we investigated the classification performance achieved with such representations of dimensions 2, 3, and 5, using the following methods.

*Principal component analysis (PCA)*: PCA is an orthogonal linear transform that maps the original feature space to a new set of orthogonal coordinates or principal components [34]. This transform is defined in such a manner that the first principal component accounts for highest global variance, and subsequent principal components account for decreasing amounts of variance. The corresponding low-dimension representations of the SIM-derived geometric feature vectors can be determined by including the appropriate number of principal components (2, 3 or 5 in this study).

*Sammon’s mapping*: Sammon’s mapping establishes a point mapping relationship between high-dimension feature vectors and a low-dimension space so that inter-point distances in the high-dimension space approximate the corresponding inter-point distances in the low-dimension space [35].

Let *X*_*i*_, *i* = 1, 2, … *N*, represent a set of high-dimension feature vectors and *Y*_*i*_, *i* = 1, 2, … *N*, their corresponding low-dimension representations. The cost function E, which represents how well the low-dimension representations *Y*_*i*_ represent the feature vectors *X*_*i*_, is given by—

$$\begin{array}{c} E=\frac{1}{\sum_{i<j}D_{ij}}\sum_{i<j}^{N}\frac{{\left(D_{ij}-d_{ij}\right)}^{2}}{D_{ij}}, \end{array}$$ (2)

where the distance between any two points *X*_*i*_ and *X*_*j*_ is represented by *D*_*ij*_, and the distance between any two points *Y*_*i*_ and *Y*_*j*_, by *d*_*ij*_. A steepest descent procedure is used for minimizing E. The implementation of this algorithm was taken from the self-organizing map (SOM) toolbox for MATLAB [41].

*t-distributed stochastic neighbor embedding (t-SNE)*: Stochastic Neighbor Embedding (SNE) converts Euclidean distances between high-dimension texture feature vectors into conditional probabilities representing similarities; the closer the feature vectors, the higher the similarity [36]. Once conditional probability distributions are established for both the high-dimension feature vectors and their corresponding low-dimension representations, the goal of the algorithm is to minimize the mismatch between the two.

Let *X*_*i*_, *i* = 1, 2, … *N*, represent a set of high-dimension feature vectors and *Y*_*i*_, *i* = 1, 2, … *N*, their corresponding low-dimension representations. Let *p*_*j*∣*i*_ be the condition probability that *X*_*i*_ selects *X*_*j*_ as a neighbor, assuming that neighbors were picked in proportion to their probability density under a Gaussian centered at *X*_*i*_. Similarly, *q*_*j*∣*i*_ is the conditional probability in the low-dimension space. Minimizing the difference between *p*_*j*∣*i*_ and *q*_*j*∣*i*_ is achieved through minimization of the sum of Kullback-Leibler (KL) divergences over all feature vectors using a gradient descent method. The cost function is given by—

$$\begin{array}{c} E=\sum_{i}KL(P_{i}\left|\right|Q_{i})=\sum_{i}\sum_{j}p_{j|i}\log\frac{p_{j|i}}{q_{j|i}}\;, \end{array}$$ (3)

where *P*_*i*_ represents the conditional probability distribution over all other feature vectors given *X*_*i*_, and *Q*_*i*_ represents the conditional probability distribution over all other low-dimension representations given *Y*_*i*_.

t-SNE was developed as an improvement over SNE to further simplify cost function optimization and overcome the so-called crowding problem inherent to SNE [36]. Details pertaining to this algorithm and its cost function minimization can be found in [36], and a review of the algorithm can be found in [23, 37]. The t-SNE implementation used in this study was taken from the dimension reduction toolbox for MATLAB [42]. t-SNE has several free parameters, such as the degrees of freedom of the t-function, the number of iterations for which the cost function optimization is processed and perplexity, which can be defined as a smooth measure of the effective number of neighbors. All parameters were defined through default settings provided by the toolbox except for perplexity, which was optimized in the supervised learning step describe later.

*Exploratory Observation Machine (XOM)*: As described in [43–45], XOM maps a finite number of data points *X*_*i*_ in a high-dimension space of dimension *D* to target points *Y*_*i*_ in the low-dimension embedding space of dimension *d*.

The initial setup of XOM involves—(1) defining the topology of the high-dimension data in the feature space through computation of distances *d*(*X*_*i*_, *X*_*j*_) between feature vectors *X*_*i*_, (2) defining a structure hypothesis represented by sampling vectors *S*_*k*_ in the low-dimension space, and (3) initializing output vectors *Y*_*i*_, one for each input feature vector *X*_*i*_. We use random samples from a uniform distribution for *S*_*k*_ in this study to enable occupation of the entire projection space. Once the initial setup was complete, the goal of the algorithm is to reconstruct the topology induced by the high-dimension feature vectors *X*_*i*_ through displacements of *Y*_*i*_ in the low-dimension space. Neighborhood couplings between feature vectors in the high-dimension space are represented by a cooperativity function *h*, which was modeled in this study as a Gaussian—

$$\begin{array}{c} h\left(X_{i},X^{'}\left(S\left(t\right)\right),\sigma \left(t\right)\right)=e^{-\frac{{\left(X_{i}-X^{'}\left(S\left(t\right)\right)\right)}^{2}}{2\sigma^{2}\left(t\right)}}. \end{array}$$ (4)

Here, *X* ^′^(*S*(*t*)) represents the *best-match* for a input feature vector *X*_*i*_. For a randomly selected sampling vector S, the *best-match* feature vector *X* ^′^ is identified by the criterion: ∣∣*S* − *Y* ^′^∣∣ = *min*_*i*_∣∣*S* − *Yi*∣∣. Once the *best-match* feature vector is identified, the output vectors *Y*_*i*_ are incrementally updated by a sequential adaptation step according to the learning rule

$$\begin{array}{c} Y_{i}\left(t+1\right)=Y_{i}\left(t\right)+\epsilon \left(t\right)h\left(X_{i},X^{'}\left(S\left(t\right)\right),\sigma \left(t\right)\right)\left(S\left(t\right)-Y_{i}\left(t\right)\right). \end{array}$$ (5)

where *t* represents the iteration step, *ϵ*(*t*) is the learning rate and *σ*(*t*) is a measure of neighborhood width taken into account by the cooperativity function *h*. In this study, both *ϵ*(*t*) and *σ*(*t*) are changed in a systematic manner depending on the number of iterations by an exponential decay annealing scheme [45]. The algorithm is terminated when either the cost criterion is satisfied, or the maximum number of iterations is completed. The above sequential learning rule can be interpreted as a gradient descent step on a cost function for XOM, whose formal derivation can be found in [37]. The final position of *Y*_*i*_ represents the low-dimension representations of the high-dimension feature vectors.

We note three free parameters in this algorithm—(1) the learning parameter *ϵ* (2) the neighborhood parameter *σ*, and (3) the total number of iterations. As with t-SNE, default settings were specified for *ϵ* and number of iterations while *σ* was optimized in the supervised learning step describe later.

### Out-of-Sample Extension

Since feature reduction through dimension reduction was restricted in its application to the training data alone, the test data were *out-of-sample* points. To obtain their corresponding low-dimension representations, the training set of high-dimension points *X*_*i*_ and their corresponding known low-dimension representations *Y*_*i*_ were used to define a mapping *F* such that *Y*_*i*_ = *F*(*X*_*i*_). This mapping *F* was then used to determine the low-dimension representations of the test set.

The goal of out-of-sample extension in this context was to create or approximate the mapping *F*. For a high-dimension feature vector *X* whose low-dimension representation is unknown, *F* can be treated as an interpolating function of the form

$$\begin{array}{c} F\left(X\right)=\sum_{i=1}^{N}a_{i}\left(X\right)Y_{i}\;, \end{array}$$ (6)

where *a*_*i*_ are the weights that define the interpolating function. We investigated two approaches to defining these weights.

*Shepard’s Interpolation*: This technique implements an inverse distance weighting approach in defining *a*_*i*_ described previously in [38], i.e.,

$$\begin{array}{c} a_{i}\left(X\right)=\frac{1/{d(X,X_{i})}^{p}}{\sum_{j=0}^{N}1/{d(X,X_{j})}^{p}}. \end{array}$$ (7)

The power parameter *p* controls how points at different distances from *X* contributed to the computation of *F*(*X*).

*Generalized Radial Basis Function Neural Network Function Approximation (GRBF-FA)*: As an alternative to Shepard’s interpolation, the mapping *F* was approximated using a generalized radial basis function neural network. The weights *a*_*i*_ were defined as,

$$\begin{array}{c} a_{i}\left(X\right)=\frac{e^{-\frac{{\left(X-X_{i}\right)}^{2}}{2\rho^{2}}}}{\sum_{j=0}^{N}e^{-\frac{{\left(X-X_{j}\right)}^{2}}{2\rho^{2}}}}\;, \end{array}$$ (8)

which represented the activity of the hidden layer of the radial basis function network. The *ρ* parameter controlled the shape of the radial basis function kernel, and defined the neighborhood of feature vectors that contributed to the computation of *F*(*X*).

Of the dimension reduction techniques investigated in this study, PCA was a special case that allowed for direct mapping of out-of-sample points into the low-dimension space and did not require any special out-of-sample extension.

We would also like to note here that such non-linear dimension reduction and out-of-sample extension techniques have free parameters which must be specified. While the typical approach is to optimize such parameters using different quality measures [35, 46, 47], we instead identified values for such free parameters that provided the best separation between the healthy and osteoarthritic classes of patterns, through cross-validation-based optimization conducted in the supervised learning step. We feel that our approach is justified since the best way to evaluate the quality of a lower-dimension projection is still unclear and under debate [46], and the end goal for dimension reduction in our study is classification and not visualization.

### Feature Reduction—Feature Selection

Feature selection involves identifying a subset of features from the input feature space that makes the most relevant contribution to separating the two different classes of feature vectors in the supervised learning step. This study used mutual information analysis to identify a subset of features from the high-dimension feature vectors that best contributed to the pattern classification task.

Mutual information (MI) is a measure of general independence between random variables [19]. For two random variables *X* and *Y*, MI is defined as—

$$\begin{array}{c} I(X,Y)=H(X)+H(Y)-H(X,Y), \end{array}$$ (9)

where entropy *H*(⋅) measures the uncertainty associated with a random variable. MI *I*(*X*, *Y*) estimates how the uncertainty of *X* is reduced when *Y* has been observed. If *X* and *Y* are independent, their MI is zero.

For the dataset of ROIs used in this study, the MI between between texture feature *f*_*s*_, which is the feature stored in the *s* ^*th*^ dimension of feature vector *f*, and the corresponding class labels *y* was calculated by approximating the probability density function of each variable using histograms *P*(⋅)—

$$\begin{array}{c} I\left(f_{s},y\right)=\sum^{n_{c}}\sum^{n_{f}}P\left(f_{s},y\right)\log_{2}\frac{P(f_{s},y)}{P\left(f_{s}\right)P\left(y\right)}\;, \end{array}$$ (10)

Here, the number of classes *n*_*c*_ = 2 was used; the number of histogram bins for the texture features *n*_*f*_ was determined adaptively according to

$$\begin{array}{c} n_{f}=\log_{2}N+1+\log_{2}\left(1+\kappa \sqrt{N/6}\right)\;, \end{array}$$ (11)

where *κ* is the estimated kurtosis and *N* the number of ROIs in the data set [20].

Once the mutual information between each feature of the original feature set and the corresponding class labels was computed, those features with the highest mutual information were selected for subsequent classification. In this study, we investigated the classification performance achieved with 2, 3 and 5 features, as selected from the original feature set using mutual information criteria. To maintain training-test separation, the best features of the texture feature vectors were selected by evaluating the mutual information criteria of the training data alone.

### Classification

The extraction of texture features and subsequent feature reduction was followed by a supervised learning step where the chondrocyte patterns were classified as healthy or osteoarthritic. In this work, support vector regression (SVR) with a linear kernel was used for the machine learning task [40]. The SVR implementation was taken from the libSVM library [48].

Owing to the practical limitations imposed by the small size of the patient population used in this study, we specified the following patient constraints to the supervised learning step—(1) ROIs from the same patient were not simultaneously used in both training and test sets, and (2) the same number of ROIs were used from every patient to ensure that the classifier did not get over-trained on patterns from a specific patient. Based on these constraints, each iteration of the supervised learning step involved randomly sub-sampling 200 ROIs from each of the five patients and randomly designating one each of the healthy and osteoarthritic subjects as the test set (the other samples comprised the training set). Such a strategy ensured that training sets used in different iterations of supervised learning were not identical despite patient constraints.

In the training phase, models were created from labeled data by employing a random sub-sampling cross-validation strategy where the training set was further split into 70% training samples and 30% validations samples. The purpose of the training phase was to determine the optimal parameters for the classifier, dimension reduction and out-of-sample extension algorithms that best captured the boundaries between the two classes of VOIs. The free parameters for the classifier used in this study were the cost parameter for SVR. Then, during the testing phase, the optimized classifier predicted the class of VOIs in the independent test set. A receiver-operating characteristic (ROC) curve was generated and used to compute the area under the ROC curve (AUC) which served as a measure of classifier performance on the independent test set. This process was repeated 50 times resulting in an AUC distribution for each feature set.

### Statistical Analysis

A Wilcoxon signed-rank test was used to compare two AUC distributions corresponding to different texture features. Significance thresholds were adjusted for multiple comparisons using the Holm-Bonferroni correction to achieve an overall type I error rate (significance level) less than *α* (where *α* = 0.05) [49, 50].

Texture, feature reduction, classifier and statistical analysis were implemented using Matlab 2010a (The MathWorks, Natick, MA).

## Results

### Evaluating Different Out-of-Sample Extension Methods

**Fig 4:**
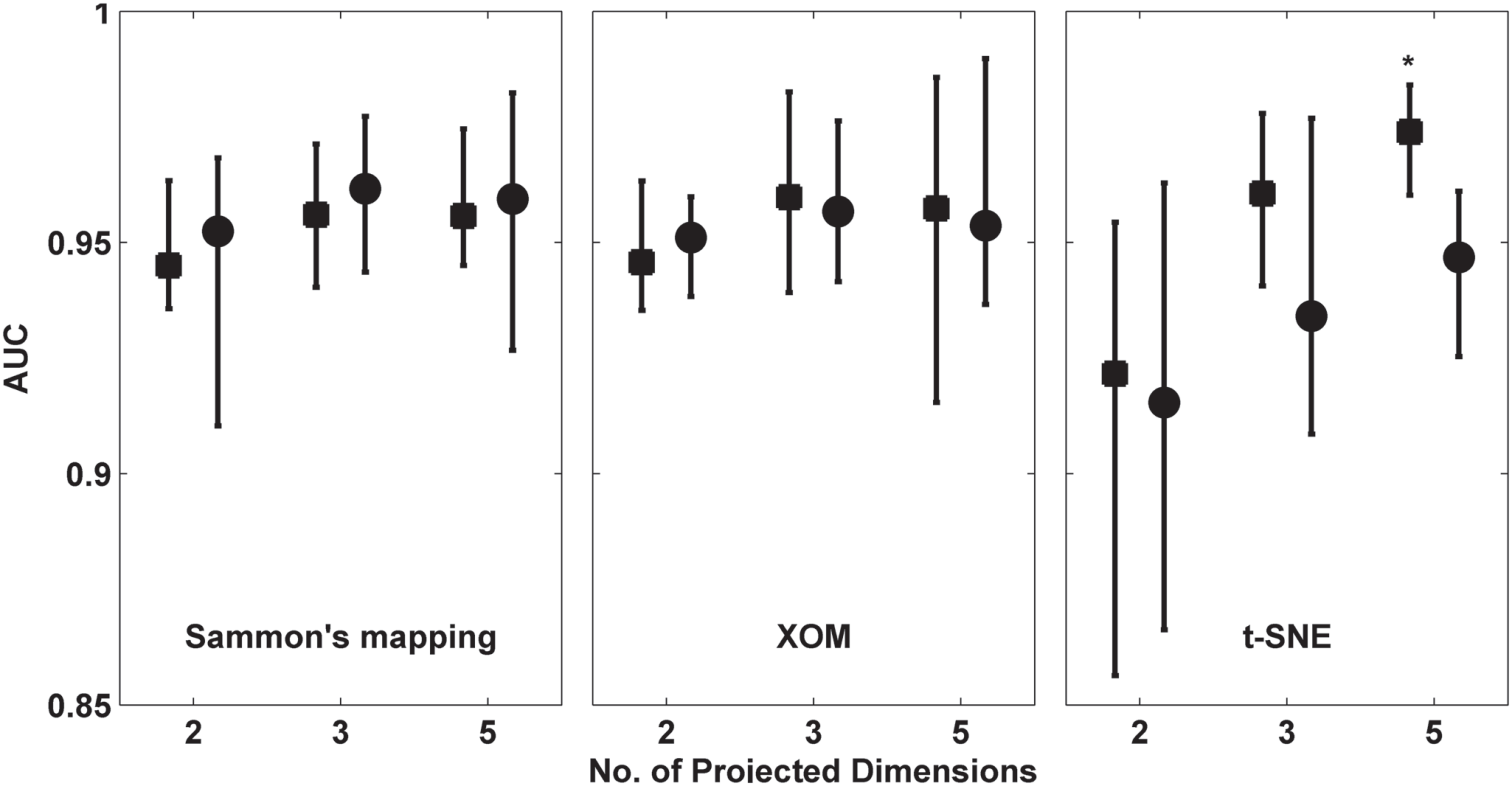
Comparison of classification performance in AUC (mean ± std) achieved with Sammon’s mapping, XOM and t-SNE algorithms when used in conjunction with different out-of-sample extension techniques, i.e. Shepard’s interpolation (square) and GRBF function approximation (circle). For each distribution, the central mark corresponds to the median and the edges are the 25^*th*^ and 75^*th*^ percentile. Comparisons where the performance achieved with Shepard’s interpolation were significantly better than those with GRBF-FA (*p* < 0.05) are marked with an asterisk.

Fig. 4 shows the classification performance achieved with the SIM-derived geometric feature vectors when processed with Sammon’s mapping, XOM and t-SNE in conjunction with the two out-of-sample extension methods outlined earlier. No significant differences in performance are observed between Shepard’s interpolation and GRBF-FA for both Sammon’s mapping and XOM. However, with t-SNE, a significant improvement in performance was noted with Shepard’s interpolation over GRBF-FA for 5-D projections of the original vectors (*p* < 0.05).

### Comparing Dimension Reduction, Feature Selection and No Feature Reduction

Table 1 shows the classification performance achieved with different feature reduction strategies pursued in this study. For each algorithm, the performance achieved with reduced feature sets of dimensions 2, 3 and 5 were evaluated and compared. For Sammon’s mapping, XOM and t-SNE, Shepard’s interpolation was used to obtain reduced feature representations of the independent test set.

**Table 1:** Classification performance achieved with different feature reduction techniques.

Algorithm	Projected Dim.	AUC
PCA	2	0.97 ± 0.02
	3	0.97 ± 0.02
	5	0.97 ± 0.02
Sammon’s Mapping	2	0.94 ± 0.03
	3	0.96 ± 0.02
	5	0.96 ± 0.02
XOM	2	0.95 ± 0.02
	3	0.96 ± 0.03
	5	0.94 ± 0.06
t-SNE	2	0.90 ± 0.09
	3	0.94 ± 0.08
	5	0.97 ± 0.02
Mutual Information	2	0.97 ± 0.01
	3	0.97 ± 0.02
	5	0.95 ± 0.02
none	9	0.96 ± 0.02

Classification performance in AUC (mean ± std) achieved with different dimension reduction and feature selection techniques investigated in this study. The last row shows the performance achieved when no feature reduction algorithm is applied, i.e. the original feature set is used for the classification task.

The classification performance achieved with the original 9-D SIM-derived geometrical feature set, i.e. with no feature reduction strategy applied, was 0.96 ± 0.02. When reducing this feature set to a 2-D representation, comparable classification was achieved by PCA (dimension reduction) and mutual information (feature selection). Other dimension reduction strategies such as Sammon’s mapping, XOM and t-SNE were significantly outperformed (*p* < 0.05). However, for 3-D and 5-D representations, all dimension reduction and feature selection strategies yielded a comparable classification performance to the original feature set.

## Discussion

Feature reduction strategies such as dimension reduction or feature selection have been previously proposed in computer-aided diagnosis (CADx) applications for obtaining efficient representations of large feature sets extracted from patterns of interest on medical images [20–25]. One advantage of identifying a reduced feature set representation of a large feature set is the reduction in processing time of the supervised learning step, as noted in this study (9-D: 6.03s, 5-D: 5.23s, 3-D: 4.88s, 2-D: 4.79s). However, the primary purpose of feature reduction in studies with finite datasets with limited patient cohort (or number of ROIs) is to prevent over-training, which stems from using too many features to describe too few ROIs, also known as the so-called curse of dimensionality [19]. This study evaluates the impact of incorporating such algorithms in the process of extracting feature sets that characterize chondrocyte organization in the radial zone of the cartilage matrix, as visualized on PCI-CT, for purposes of automated classification. The motivation for our work stems from previous demonstrations of PCI-CT’s ability to visualize structural details of the human patellar cartilage matrix with high spatial resolution [15]. This makes cartilage imaging with PCI-CT a suitable target for soft tissue characterization with novel texture features [17, 18]. Given that such textural characterization usually yields a large feature set, it is important to obtain efficient representations of these extracted features through some feature reduction strategy.

In this study, we demonstrated a new CADx methodology for automated classification of healthy and osteoarthritic cartilage that incorporates dimension reduction into CADx while simultaneously maintaining a strict separation between training and test sets. This differentiates our study from previous attempts at using dimension reduction in CADx where such algorithms where applied to the entire dataset [23, 24]. Such implementation compromises the integrity of the independent test set, since feature vectors belonging to the training and test sets are free to interact and influence the computation of low-dimension representations. Our new methodology maintains the required training-test set separation by applying dimension reduction to the training data alone; corresponding low-dimension representations for the test set are obtained through out-of-sample extension techniques. Our methodology explored the integration of dimension reduction techniques such as PCA, Sammon’s mapping, XOM and t-SNE in conjunction with out-of-sample extension techniques such as Shepard’s interpolation and GRBF-FA for reducing the size of the originally extracted feature set.

**Fig 5:**
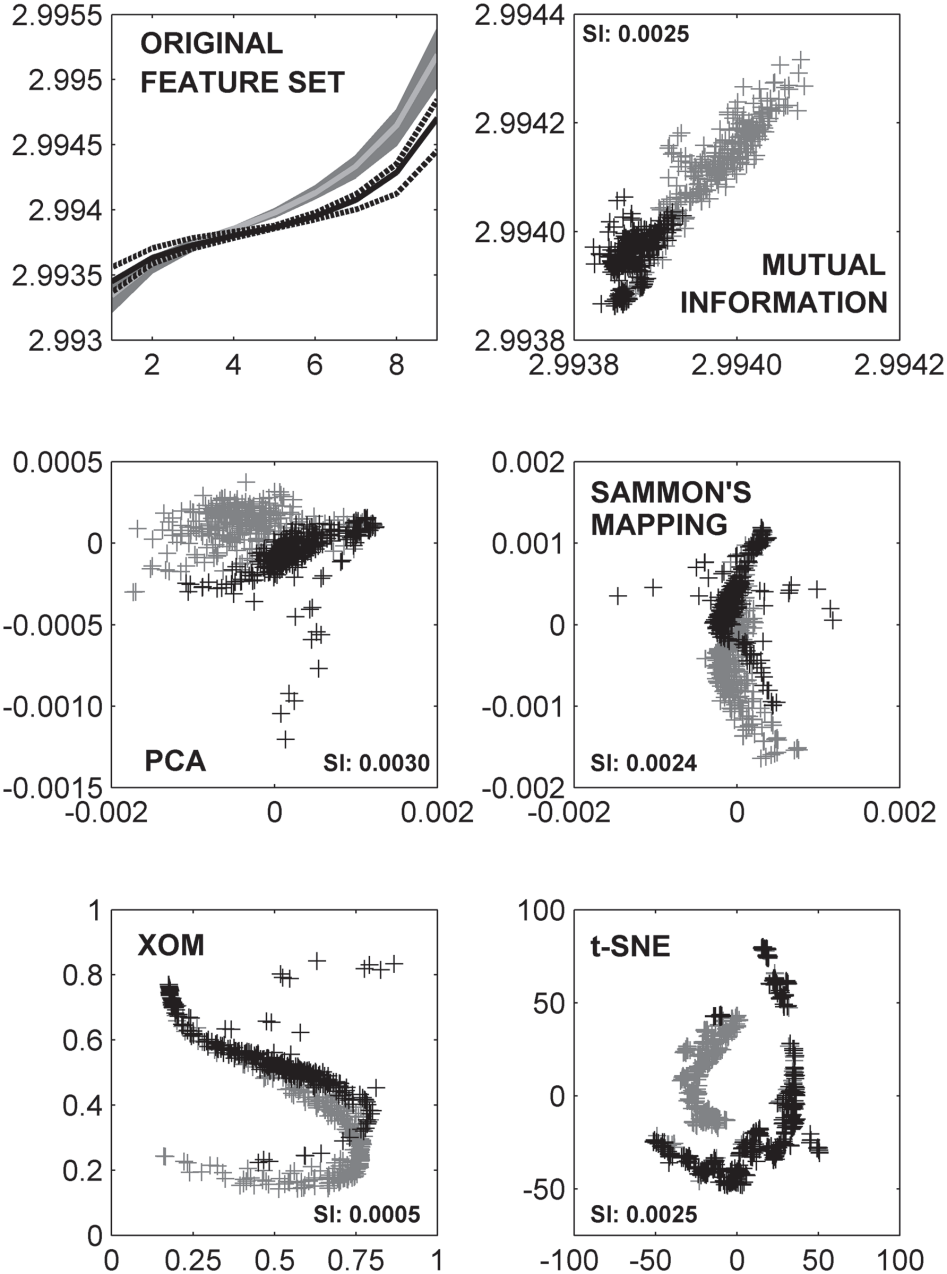
An exploration of the SIM feature space. In all plots, representations of feature vectors extracted from healthy VOIs are colored gray, those from osteoarthritic VOIs are colored black. Cluster separation is quantified with Dunn’s separation index (SI), and specified in each plot. (Top Left) SIM feature vectors extracted from normal diseased ROIs area. The distribution of curves corresponding to each class is enclosed by the 25^*th*^ and 75^*th*^ percentile curves, the solid line represents the median curve. (Top Right) Plotting the 2-D reduced feature representation of the SIM feature set as obtained through evaluation of mutual information criteria. (Middle) Plots of 2-D projections of the SIM feature set obtained with PCA (left) and Sammon’s mapping (right). (Bottom) Plots of 2-D projections of the SIM feature set obtained with XOM (left) and t-SNE (right). As seen, here all feature reduction techniques yield discernible clusters of healthy and osteoarthritic VOIs, but with varying degrees of overlap.

Our results suggest that the high classification performance achieved with SIM-derived geometrical features (0.96 ± 0.02) can be maintained while substantially reducing the size of the original feature set. The fact that no statistically significant deterioration in performance is observed with the original feature set suggests room for further inclusion of other features when necessary. We specifically note that both dimension reduction through PCA and feature selection with mutual information were able to yield 2-D representations of the 9-D SIM feature set extracted from the patellar cartilage ROIs without compromising the classification performance achieved. Non-linear dimension reduction techniques such as Sammon’s mapping, XOM and t-SNE exhibited a small but significant deterioration in performance for 2-D projections of the original feature set, but exhibited comparable performance for 3-D and 5-D projections. The high classification performance noted with both the original feature set as well as the reduced feature set representations obtained with different techniques is further illustrated by Fig. 5, where distinct clusters of the healthy and osteoarthritic features are observed, albeit with poor separation as indicated by the corresponding Dunn’s separation index [51]. Note that differences between the two clusters are only emphasized in the machine learning step; such visualizations are for exploratory analysis of the feature space only.

The high degree of compaction achieved by such techniques, i.e. reducing 9-D feature set to 2-D or 3-D, suggests tremendous potential for future application in computational tools for radiologists. As an example, content-based image retrieval (CBIR) could retrieve prior cases with similar patterns based on an annotated pattern in the current study. Such CBIR tools could rely on matching feature sets extracted from the current study to those previously extracted from other studies and stored in some database. In such a scenario, its not surprising that the computational efficiency (in terms of processing speed, memory usage etc) improves when the feature sets are smaller in size. As long as such feature reduction approaches are only used in such ancillary support tools for radiologists in a clinical setting and not interpreted directly for evaluation of clinical findings, we anticipate minimal impact on clinical work flow in terms of information loss.

While we observe no significant differences in performance when using dimension reduction or feature selection for reducing the size of the original feature set in this study, their advantages and disadvantages are worth highlighting. Feature selection explicitly exclude features and results in a loss of information. Such losses could be relatively minimized in dimension reduction strategies where all features in the original set contribute to the final low-dimension representations. This has been previously observed when attempting to compact large feature sets (eg. 100-D) into very small representations (2-D/3-D) [25]. However, feature selection allows for identification of features that were selected as part of the reduced set. Dimension reduction results in the creation of new features, and the contributions of the original features to the reduced feature set is not readily interpretable. This will likely serve as an important criterion to consider while deciding upon which feature reduction strategy to pursue for a specific problem.

One must also note an inherent concern in integrating dimension reduction in its current form into CADx despite the promising results reported in this study. CADx aims to best separate different classes of feature vectors while dimension reduction attempts to best represent high-dimension data in a low-dimension space through some optimization paradigm (preservation of distances, similarities, topologies etc). These are essentially two independent optimization tasks with goals that are not guaranteed to align. One may explore dimension reduction techniques that incorporate some form of class discrimination while computing the low-dimension representations of the high-dimension feature vectors. Supervised dimension reduction variants of learning vector quantization approaches such as the neighbor retrieval visualize (NeRV) algorithm [46], generalized matrix learning vector quantization (GMLVQ) [52], or limited rank matrix learning vector quantization (LiRAM LVQ) [53], would be better suited to integration with our CADx methodology proposed in this study.

Finally, we acknowledge some limitations with the current study. To facilitate comparisons between different feature reduction algorithms, we arbitrarily fixed the sizes of the reduced feature sets to 2, 3 and 5. One could instead optimize for the smallest number of features that either yield the best classification performance or maintain the performance of the original feature set. A small number of patients served as donors of the cartilage specimens for PCI-CT imaging and as a result, the classifier could be over-trained to the limited variations of healthy and osteoarthritic patterns found in these subjects. Future studies should include a larger patient cohort to ensure that the classifier is trained with a potentially larger variation of healthy and osteoarthritic patterns.

## Conclusion

We demonstrate a CADx methodology with integrated feature reduction using either dimension reduction or feature selection in the research context of classifying healthy and osteoarthritic patellar cartilage annotated on PCI-CT images. We specifically outline a method to integrate dimension reduction in CADx while concurrently maintaining a strict training-test set separation required for supervised learning components. Our results suggest that both feature selection and dimension reduction could maintain the performance of the original pattern characterizing feature set while achieving a high degree of feature compaction. We hypothesize that such an approach would have significant practical advantages in a clinical setting as low-dimension representations of large feature sets extracted from annotated patterns can contribute to improved efficiency in terms of storage, processing speed etc. However, larger controlled trials need to be conducted in order to further validate the clinical applicability of our method.

## Supporting Information

S1 Dataset High-dimensional SIM-derived geometrical feature vectors and corresponding label data for the PCI-CT VOIs used in this manuscript. (MAT)
